# Supplemental Silicon and Boron Alleviates Aluminum-Induced Oxidative Damage in Soybean Roots

**DOI:** 10.3390/plants13060821

**Published:** 2024-03-13

**Authors:** Shuwei Wang, Haijing Cheng, Yunmin Wei

**Affiliations:** 1 College of Life Sciences and Oceanography, Shenzhen University, Shenzhen 518060, China; shuweiwang_21@163.com (S.W.); 13644654490@163.com (H.C.); 2 College of Optoelectronic Engineering, Shenzhen University, Shenzhen 518060, China

**Keywords:** soybean (*Glycine max* L.), silicon, boron, aluminum, oxidative damage, citric acid

## Abstract

Aluminum (Al) toxicity in acidic soils is a major abiotic stress that negatively impacts plant growth and development. The toxic effects of Al manifest primarily in the root system, leading to inhibited root elongation and functionality, which impairs the above-ground organs of the plant. Recent research has greatly improved our understanding of the applications of small molecule compounds in alleviating Al toxicity. This study aimed to investigate the role of boron (B), silicon (Si), and their combination in alleviating Al toxicity in soybeans. The results revealed that the combined application significantly improved the biomass and length of soybean roots exposed to Al toxicity compared to B and Si treatments alone. Our results also indicated that Al toxicity causes programmed cell death (PCD) in soybean roots, while B, Si, and their combination all alleviated the PCD induced by Al toxicity. The oxidative damage induced by Al toxicity was noticeably alleviated, as evidenced by lower MAD and H_2_O_2_ accumulation in the soybean roots treated with the B and Si combination. Moreover, B, Si, and combined B and Si significantly enhanced plant antioxidant systems by up-regulating antioxidant enzymes including CAT, POD, APX, and SOD. Overall, supplementation with B, Si, and their combination was found to alleviate oxidative damage and reduce PCD caused by Al toxicity, which may be one of the mechanisms by which they alleviate root growth inhibition due to Al toxicity. Our results suggest that supplementation with B, Si, and their combination may be an effective strategy to improve soybean growth and productivity against Al toxicity.

## 1. Introduction

Aluminum (Al) is the third most abundant element on Earth and is a widely available metal. It constitutes about 8.2% of the Earth’s crust, following only oxygen and silicon in terms of abundance. In neutral or nearly neutral soil, Al is present in the form of insoluble oxides or aluminosilicates, typically exhibiting a low solubility, which poses no toxic threat to plants. However, in acidic soils with a pH below 5.5, Al mainly exists in the form of Al(OH)^2+^, Al(OH)^+^, and Al(H_2_O)^3+^, which can be solubilized and released into the soil. Al ions (mainly Al^3+^) are highly toxic to most agriculturally cultivated plants [1,2]. In acidic soils, high concentrations of aluminum ions can interfere with plant growth and development by damaging root systems. This interference significantly inhibits root elongation, reduces water and mineral nutrient absorption, and disrupts various physiological processes. Additionally, numerous studies have reported that aluminum induces the rapid production and accumulation of reactive oxygen species (ROS), severely impacting plant cell metabolism, and accelerates Al-induced programmed cell death (PCD) of plants [3,4,5]. Al-induced lipid peroxidation results in the production and accumulation of malondialdehyde (MDA), which disrupts membrane functions. This effect has been reported in various plants, including tomato (*Solanum lycopersicum* L.), rice (*Oryza sativa* L.), soybean (*Glycine max* L.), tobacco (*Nicotiana tabacum* L.), and pea (*Pisum sativum* L.) [6,7,8]. Despite their deleterious effects, ROS play a dual role in plants, as they also serve as important signaling molecules to activate the expression of antioxidant mechanisms to alleviate Al stress [9,10,11]. Additionally, some plants stimulate organic acid (e.g., malate, citrate) exudation from their root surface, thereby chelating and immobilizing the phytotoxic Al^3+^ in the rhizosphere [12,13,14,15]. For instance, soybeans can relieve Al stress by secreting citric acid [16,17].

Furthermore, recent studies have demonstrated that silicon (Si) supplementation can potentially mitigate Al toxicity in plants [18,19,20,21]. The second most abundant element in the Earth’s crust is silicon, which is second only to oxygen in content and constitutes about 27.7% of the Earth’s mass [22]. In soil solutions, Si exists as silicic acid (Si[OH]_4_) and is readily absorbed by the roots of higher plants [23]. Although Si is not a macronutrient for most plants, it plays an important role in plant responses to various abiotic stresses, including Al toxicity [24,25]. Si can alleviate Al toxicity in plants via multiple mechanisms, such as reducing the deposition of Al in the cell wall, increasing antioxidants, forming Si–Al complexes, and enhancing the rhizosphere pH [26,27,28,29]. The application of Si to plants exposed to Al stress has been shown to improve the growth and yield of various crops, such as rice, maize, and soybean. These results highlight the potential of Si application in sustainable agriculture [27,30].

Boron (B) is beneficial for plant growth and is an indispensable micronutrient [31,32]. Several studies have shown that B plays a major role in mitigating Al toxicity in many plants, such as *Citrus grandis*, peas, rape seedlings, rice, and so on [33,34,35,36,37]. Supplementation with B effectively reduced the accumulation of Al in cell walls and reduced oxidative damage to roots by modulating their cell wall composition and structure [36,37]. Recent research has reported that B supplementation can alleviate the oxidative stress of Al on citrus roots by reducing the hydrogen peroxide content and lipid peroxidation (indicated by MDA content) [35,37].

Soybean is an important cash crop that is widely cultivated worldwide and is used as a food, feed, and oilseed crop. It is a major source of proteins and edible oil [38]. The growth, biomass production, and essential physiological functions of soybean plants are significantly impaired by aluminum toxicity, thereby adversely impacting productivity [39]. Previous studies have identified Si and B as potential alleviators of Al toxicity in soybeans, but the underlying mechanisms remain poorly understood. Therefore, the present study aimed to explain the inhibitory effects of boron, silicon, and their combination on Al-induced root growth and oxidative damage in soybeans. In addition, the interventions were hypothesized to alleviate Al toxicity in soybeans by up-regulating the antioxidant defense system and secreting organic acids.

## 2. Results

### 2.1. Effects of B, Si, and Their Combination on Soybean Root Growth Parameters under Al Stress

**Figure 1:**
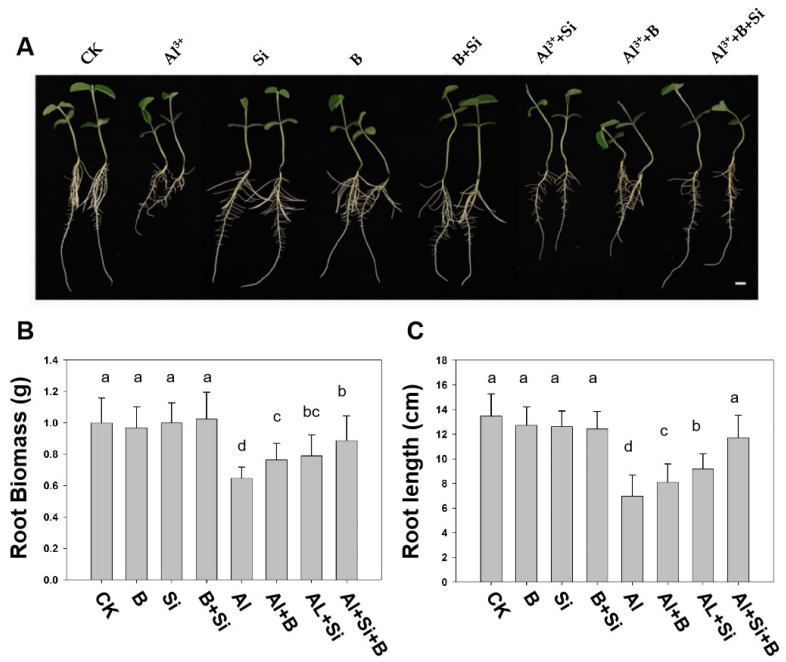
Effects of B, Si, and their combination on soybean root growth parameters under Al stress. (**A**) Symptoms, (**B**) root biomass, (**C**) root length. Here, (1) CK (without Al, B, and Si), (2) + B (16.3 µM H_3_BO_3_), (3) + Si (1.5 mM Na_2_SiO_3_), (4) B + Si (16.3µ M H_3_BO_3_ + 1.5 mM Na_2_SiO_3_), (5) Al (50 µM AlCl_3_), (6) Al + B (50 µM AlCl_3_ + 16.3 µM H_3_BO_3_), (7) Al + Si (50 µM AlCl_3_ + 1.5 mM Na_2_SiO_3_), and (8) Al + B + Si (50 µM AlCl_3_ + 1.5 mM Na_2_SiO_3_ + 16.3 µM H_3_BO_3_). Scale bar, 1 cm. Bars indicate the means of three replicates ± SD. The different letters (a, b, c, d) in each sub-figure represent significant differences at *p* < 0.05.

As shown in Figure 1, B and Si alone and their combination (B + Si) exerted no effect on soybean root growth in the absence of Al stress compared to the non-treated plants (CK). However, upon exposure to 50 µM AlCl_3_, Al toxicity significantly reduced the root biomass and length of soybeans compared to CK. However, the addition of B and Si significantly alleviated the Al toxicity-induced inhibition of root elongation. Compared to the Al treatment, B and Si treatments increased root length by 16.3% and 31.7%, respectively, and root biomass by 18.5% and 22.4%, respectively. Moreover, the Al + B + Si group demonstrated a significant increase in root length of 68.3% compared to the plants only exposed to Al, which effectively recovered to CK levels. Furthermore, the root biomass of B and Si co-treated plants exposed to Al toxicity significantly increased by 37.4% compared to Al-treated soybean seedlings.

### 2.2. Effect of B, Si, and Their Combination on Al Toxicity-Induced PCD and Al Accumulation in Soybean Roots

**Figure 2:**
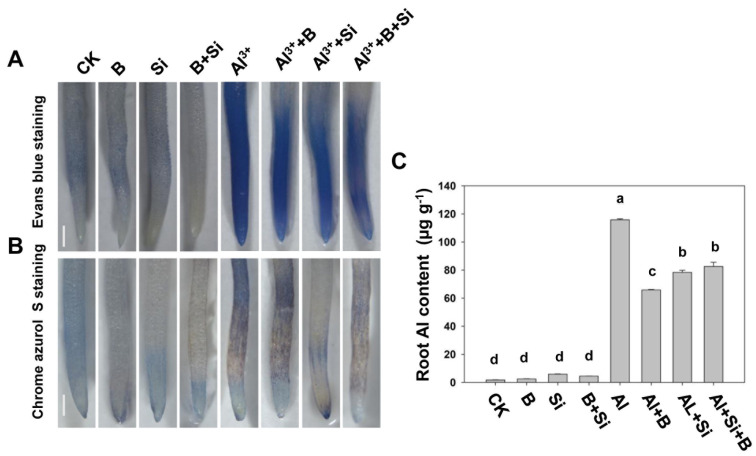
Effect of B, Si, and their combination on Al toxicity-induced PCD and Al^3+^ accumulation in soybean roots. (**A**) Root PCD; (**B**,**C**) root Al content. The bars indicate the means of three replicates ± SD. Scale bar, 1 mm. Different letters (a, b, c, d) in each sub-figure represent significant differences at *p* < 0.05.

Treated soybean root tips were stained with Evans blue to investigate the effects of Si, B, and their combination on Al toxicity-induced programmed cell death (PCD) in soybean roots. As shown in Figure 2A, Si and B alone and their combination did not induce PCD in soybean root tips in the absence of Al stress. Upon exposure to 50 µM Al stress, significantly elevated PCD levels were observed in soybean root tips, which were significantly alleviated by B and Si treatment alone and their combination (B + Si). Al stress resulted in the accumulation of Al in root tips, which may contribute to root tip PCD. Therefore, the effects of B, Si, and their combination on Al accumulation in root tips were analyzed using Chrome Azurol S staining and the flame atomic absorption spectrophotometry (FAAS) method. The results revealed that Al stress greatly increased the accumulation of Al in roots, whereas the addition of B and Si alone and their combination reduced Al accumulation by 43.2%, 32.3%, and 28.7%, respectively (Figure 2B,C).

### 2.3. Effects of B, Si, and Their Combination on Lipid Peroxidation and H_2_O_2_ Content in Soybean Roots under Al Stress

**Figure 3:**
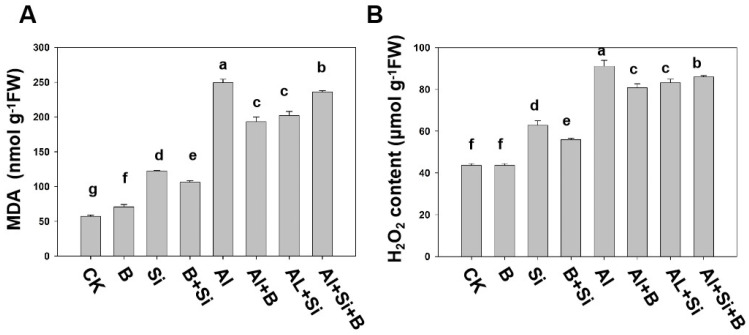
The effect of B, Si, and their combination on (**A**) lipid peroxidation (MDA) and (**B**) H_2_O_2_ modulation in soybean roots under Al-induced toxicity. The bars represent the means of three replicates ± SD. The different letters (a, b, c, d, e, f, g) in each sub-figure represent significant differences at (*p* < 0.05).

ROS-induced lipid membrane peroxidation and H_2_O_2_ production induce oxidative damage at the cellular level. The experiment results demonstrated that Al stress rapidly produced MDA and H_2_O_2_, thereby disrupting cellular structures (Figure 3). A noticeable rise in MDA levels was observed solely in the roots of soybean plants treated with Al^3+^ when compared to plants treated with Si, B, or their combination. The effects of aluminum toxicity were significantly reduced in plants treated solely with silicon or boron compared to plants treated with both silicon and boron. B and Si treatment alone displayed the lowest MDA levels in the roots, showing a reduction of 22.5% and 18.8%, respectively. In contrast, the combination-treated plants exhibited a 5.2% reduction in MDA levels compared to plants exposed to Al^3+^ alone (Figure 3A). Similarly, Al stress significantly elevated the levels of H_2_O_2_ in roots compared to plants treated with B, Si, and their combination. B, Si, and their combination significantly reduced the production and accumulation levels of H_2_O_2_ compared to Al-only treated plants, showing a reduction of 11.3%, 8.8%, and 5.6%, respectively (Figure 3B). In summary, B, Si, and their combination significantly decreased H_2_O_2_ and MDA levels induced by Al toxicity.

### 2.4. Effects of B, Si, and Their Combination on Antioxidant Activities in Soybean Roots under Al Stress

**Figure 4:**
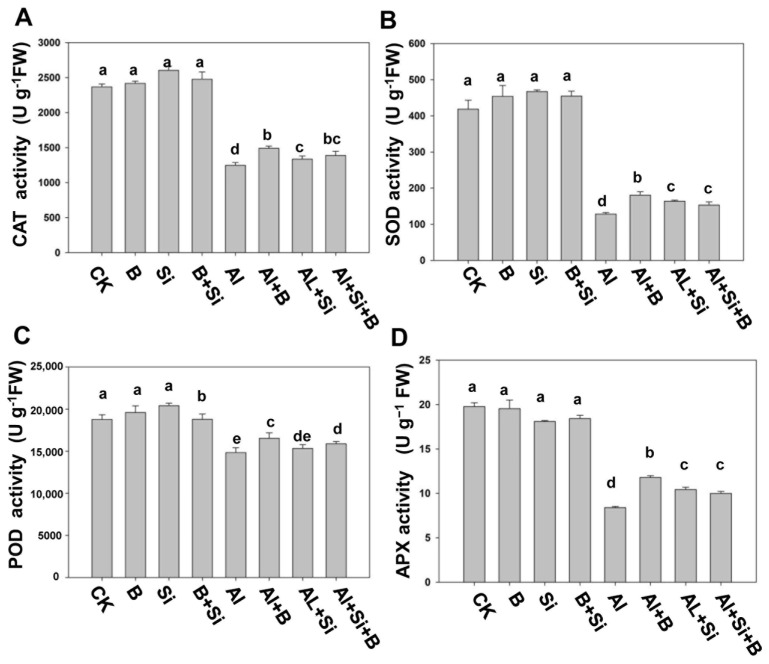
Effects of B, Si, and their combination on antioxidant activities in soybean roots under Al stress. (**A**) APX activity, (**B**) CAT activity, (**C**) POD activity, (**D**) SOD activity. The bars indicate the means of three replicates ± SD. The different letters (a, b, c, d, e) in each sub-figure represent significant differences at *p* < 0.05.

The roles of B, Si, and B + Si in mitigating Al toxicity were assessed by analyzing the activity of antioxidant enzymes (APX, CAT, POD, and SOD) in soybean roots under Al stress (Figure 4). In the absence of Al treatment, B, Si, and their combination treatment did not affect CAT activity levels in roots compared to the CK group, as shown in Figure 4B. However, the CAT activities in roots were significantly reduced when plants were exposed to Al stress. The results revealed that B, Si, and their combination significantly enhanced CAT activity by 1.2-, 1.07-, and 1.1-fold, respectively, compared to Al treatment alone. Likewise, SOD activities in roots were significantly increased in all treatments under Al stress compared to the Al treatment alone, showing an increase of 1.4-, 1.3-, and 1.2-fold for B, Si, and their combination, respectively (Figure 4D). Similarly, in terms of POD and APX mediation, Al stress significantly attenuated the activities of both POD and APX compared to the CK group and the B, Si, and B + Si treatment groups without Al (Figure 4A,C). Compared to Al treatment alone, B treatment under Al stress significantly increased root POD activity levels by 1.1-fold, followed by Si and the combination treatment, showing a 1.03- and 1.07-fold increase, respectively. As shown in Figure 4A, the APX activity demonstrated a marked increase of 1.4-fold in roots treated with Al + B. In contrast, Al + Si and Al + B + Si treatments resulted in a 1.3- and 1.2-fold increase, respectively, compared to Al treatment alone. In summary, supplementation with B and Si, as well as their combination, improved the resistance of soybean seedlings to Al stress. This was achieved by increasing the activity of antioxidant enzymes, which in turn reduced the negative effects of oxidative stress caused by Al toxicity in the soybean seedlings.

### 2.5. Effects of B, Si, and Their Combination on the Secretion of Citric Acid in Soybean Roots under Al Stress

**Figure 5:**
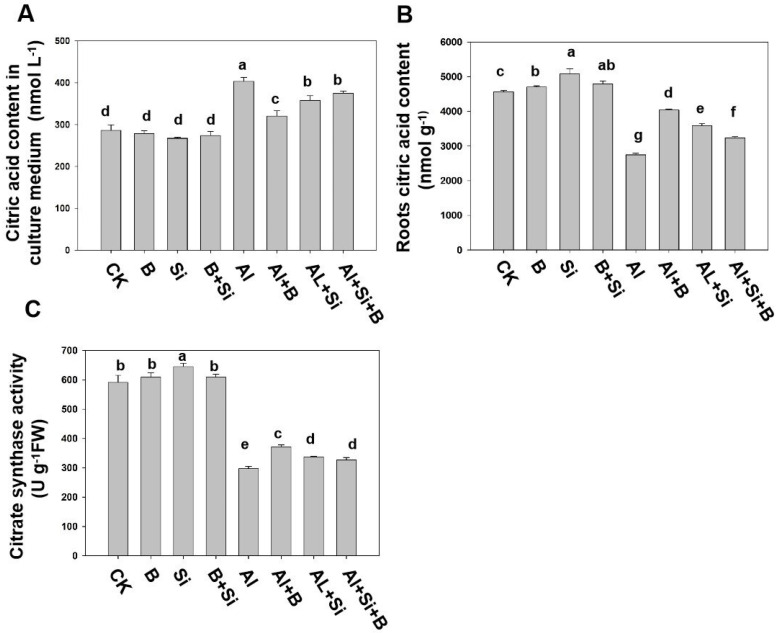
Effects of Si, B, and their combination on the secretion of citric acid in soybean roots under Al stress. (**A**) Citric acid in culture medium, (**B**) citric acid in roots, (**C**) citric synthase (CS) activity in roots. The bars indicate the means of three replicates ± SD. The different letters (a, b, c, d, e, f, g) in each sub-figure represent significant differences at *p* < 0.05.

Studies have shown that soybeans (*Glycine max*) resist Al stress by secreting citric acid. Our results are consistent with this evidence, indicating a notable rise in the concentration of citric acid in the treatment solution due to Al stress compared to plants that were not subjected to Al treatment (Figure 5A). Interestingly, compared with the control without Al treatment, B, Si, and their combination increased the level of citric acid in the treatment solution when plants were exposed to Al stress. However, B, Si, and their combination treatment showed significantly reduced citric acid levels in the treatment solution under Al stress (Figure 5A). B, Si, and their combination substantially increased citric acid levels compared to Al treatment alone (Figure 5B). Similarly, B, Si, and their combination significantly enhanced the activity of citrate synthase (CS) by 1.3-, 1.1-, and 1.1-fold, respectively, compared to Al treatment alone (Figure 5C).

## 3. Discussion

Al toxicity severely limits plant growth and productivity by negatively affecting plant physiology and metabolic processes in acidic soils [1,7,40,41]. Numerous studies have demonstrated that Al stress inhibits plant root elongation. This study revealed the inhibited root growth potential and parameters under Al toxicity (50 μM Al) (Figure 1). Several mechanisms have been proposed for plant resistance to aluminum toxicity, and it has become evident that B and Si play an indispensable role in plant tolerance to aluminum toxicity [42]. In our current study, the B + Si combination and B and Si alone significantly reversed the root growth inhibition induced by Al toxicity (Figure 1C), which is consistent with earlier reports in citrus and date palm seedlings [36]. Programmed cell death (PCD) plays an essential role in plant development and stress. Al stress has been found to induce PCD in a variety of plants, such as peanut, pea, and barley [3]. Al-induced PCD of peanut root tip cells is responsible for Al toxicity and shows a direct relationship with the suppression of root elongation caused by Al [43]. Consistent with other studies, our results indicated that Al toxicity causes PCD in soybean roots, while B, Si, and their combination alleviated the PCD induced by Al toxicity (Figure 2A). There is evidence that Al-induced PCD is closely related to ROS bursts [44]. Supplementation with B, Si, and their combination alleviated oxidative damage and thereby reduced PCD caused by Al toxicity, which may be one of the mechanisms by which they alleviate root growth inhibition due to Al toxicity. However, the specific mechanism remains to be further studied in the future.

B and Si supplementation has been hypothesized to enhance plant antioxidant defense systems, thereby mitigating Al toxicity [42,45]. As stated above, MDA and H_2_O_2_ levels were significantly elevated in soybean roots under Al stress (Figure 3A,B). The accumulation of H_2_O_2_ may enhance the production of hydroxyl radicals and thus lipid peroxidation (Figure 2A,B), which may explain the inhibitory effect of Al toxicity on root elongation. Moreover, higher root PCD levels and relative root length parallelled higher H_2_O_2_ production (Figure 1A,C, Figure 2A and Figure 3B), indicating that Al-induced oxidative bursts play an active role in the induction of PCD. Moreover, B, Si, and their combination reduced MDA and H_2_O_2_ levels and alleviated root growth inhibition under Al stress (Figure 1 and Figure 2), suggesting that B, Si, and their combination mitigates Al toxicity by reducing the accumulation of ROS [36,42,46].

Plants have developed various protective mechanisms, both enzymatic and non-enzymatic, to efficiently eliminate ROS. APX, CAT, POD, and SOD are the four key antioxidant enzymes in the enzymatic detoxification system. Our results indicate that aluminum toxicity significantly inhibited SOD activities, while the addition of B, Si, and their combination alleviated this inhibition (Figure 4D). It can be confirmed that the addition of B, Si, and their combination is related to the reduction in the accumulation of free radicals in cells and the damage to the membrane system. B, Si, and their combination can activate APX, CAT, and POD, which are the antioxidant enzymes responsible for scavenging H_2_O_2_ under Al stress (Figure 4A–C). Consistent with our findings, Pontigo et al. observed that Si significantly reduced lipid peroxidation induced by Al toxicity, and similarly, B application reduced oxidative damage to plants subjected to Al stress [42,45,46]. In summary, supplementation with B, Si, and their combination mitigated oxidative damage by reducing the accumulation of ROS and MAD and triggering the up-regulation of the antioxidant system (Figure 3 and Figure 4).

Many studies have confirmed a significant correlation between Al toxicity resistance and the level of Al-dependent release of organic acids; in particular, the exudation of citric acid from roots determines the Al resistance of the plant [1,33]. Under Al stress, the treatment solution experienced a noteworthy decline in citric acid levels when B, Si, or a combination of both were introduced, as evidenced by our results (Figure 5A). However, addition of B, Si, and their combination enhanced citric acid levels and CS activity in roots under Al stress (Figure 5B,C). This may suggest that although both B and Si are involved in regulating the metabolic pathway of citric acid synthesis in soybean roots, they do not serve as the primary mechanism for alleviating Al toxicity-induced inhibition of root growth. However, there are still many problems to be solved concerning how B, Si, and their interactions regulate the internal and external tolerance mechanisms of plants subjected to Al toxicity, especially the organic acid pathway.

Based on our results showing the positive roles of Si, B, and their combination in mitigating Al toxicity in soybeans, adding B and Si to the soil or through foliar sprays may help slow the effects of Al toxicity in soybeans. For areas where Al toxicity in the soil is a serious problem, you can consider adding B and Si amendments to increase the biological activity of the soil, thereby improving the growing environment of plants. However, acidic soil is a complex environment influenced by numerous factors, including plant species and age, necessitating further research to provide a theoretical basis for B and Si to mitigate Al toxicity. In the future, through in-depth research on the mechanism of these trace elements, their application methods and dosages in plant cultivation can be optimized to achieve the best reduction in Al toxicity. More experiments and trials are still needed to verify the molecular mechanisms and actual roles of B and Si in mitigating Al toxicity. Taken together, B and Si have potential applications in mitigating Al toxicity in plants, but further research and practice are needed to determine the best application methods and effect evaluations.

## 4. Materials and Methods

### 4.1. Plant Growth and Experimental Design

**Figure 6:**
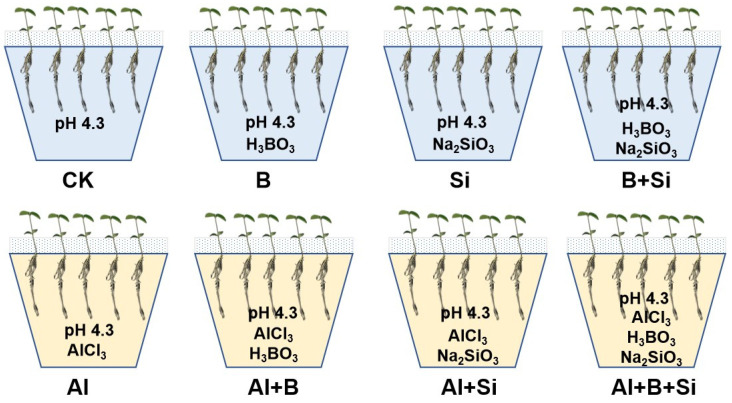
An illustrative experimental diagram explaining the eight different treatments.

Soybean seeds underwent sterilization by immersing them in a solution containing 0.5% sodium hypochlorite for a period of 15 min. The seeds were rinsed several times with distilled water, germinated on trays for 3 days, and watered with distilled water daily. After 3 days of seed germination, seedlings of uniform length with a uniform number of roots were selected and transferred to 15 L plastic pots, each with 20 seedlings, containing 25% strength Hoagland solution without Al. After true leaves emerged, seedlings with similar root lengths were transferred to solutions containing 0.5 mM CaCl_2_ at pH 4.3 and pre-treated for 1 day. The seedlings were then divided into the following eight groups: (1) CK (solutions containing 0.5 mM CaCl_2_, pH 4.3), (2) B (solutions containing 0.5 mM CaCl_2_ and 16.3 µM H_3_BO_3_, pH 4.3), (3) Si (solutions containing 0.5 mM CaCl_2_ and 1.5 mM Na_2_SiO3, pH 4.3), (4) B + Si (solutions containing 0.5 mM CaCl_2_, 16.3 µM H_3_BO_3_, and 1.5 mM Na2SiO3, pH 4.3), (5) Al (solutions containing 0.5 mM CaCl_2_ and 50 µM AlCl_3_, pH 4.3), (6) Al + B (solutions containing 0.5 mM CaCl_2_, 50 µM AlCl_3_, and 16.3 µM H_3_BO_3_, pH 4.3), (7) Al + Si (solutions containing 0.5 mM CaCl_2_, 50 µM AlCl_3_, and 1.5 mM Na_2_SiO_3_, pH 4.3), and (8) Al + B + Si (solutions containing 0.5 mM CaCl_2_, 50 µM AlCl_3_, 16.3 µM H_3_BO_3_, and 1.5 mM Na_2_SiO_3_) (Figure 6). All the solutions were prepared in distilled water. The plastic pots were prepared in triplicate and placed in a greenhouse in a randomized arrangement; fresh solutions were replaced every other day. After 7 days of treatment, the lengths and fresh weights of roots were measured, and seedling roots were carefully collected and put in a −80 °C refrigerator after quick-freezing with liquid nitrogen.

### 4.2. Root Biomass and Length Measurement

In each repetition, five plants were randomly selected, and their roots were separated and dried on paper towels for measurement using an electronic analytical balance. The length of the roots was evaluated with the help of a graduated ruler.

### 4.3. Determination of Al Content in Root

The Al content in the dried roots was analyzed. The root samples were ashed at 500 °C for 8 h and treated with 2 M HCl. Subsequently, 4 g of sodium hydroxide and 1 g of sodium peroxide was added to the mixture, which was then melted in a muffle furnace at 650 °C for 15 min. The resulting solution was filtered, and the total amount of Al was quantified by FAAS at 324.7 nm [47]. Al content was localized in roots by Chrome Azurol S staining. The roots were fixed in 2.5% (*v*/*v*) glutaraldehyde in 0.1 M sodium phosphate buffer (pH 7.2). Subsequently, the samples were stained with 0.5% Chrome Azurol S for 1 h. A positive reaction was determined by bluish staining. Images were taken using a light microscope (Axio Zoom.V16, Carl Zeiss, Oberkochen, Germany) equipped with software to capture images (AxioVision software Release 4.8, Carl Zeiss Vision).

### 4.4. Root Cell Death Detection

Al-induced cell death in the roots was assessed following Evans blue staining (0.005%, *w*/*v*). The roots were mixed and incubated with the dye for 10 min (Delphine Arbelet-Bonnin2018). Dead cells appeared with a bluish stain. Images were captured using a light microscope.

### 4.5. Lipid Peroxidation (MDA) and Hydrogen Peroxide (H_2_O_2_) Content in Soybean Roots

The extent of lipid peroxidation and the formation of MDA and H_2_O_2_ content in the roots was measured using an MDA content assay kit (Shanghai Sangon Biotech, Shanghai, China) and a commercially available Hydrogen Peroxide (H_2_O_2_) Content Assay Kit (Shanghai Sangon Biotech, Shanghai, China). Briefly, approximately 0.1 g of sample was placed into 1 mL of extraction solution and then centrifuged at 4 °C and 12,000 rpm for 10 min. Subsequently, 300 µL of supernatant was added to a reaction solution containing 0.5% thiobarbituric acid (TBA), followed by incubation at 95 °C for 30 min and centrifugation. The MDA content was determined and calculated according to the manufacturer’s instructions. Furthermore, 0.1 g of sample was placed into 1 mL of acetone. The mixture was homogenized in an ice bath and then centrifuged. Subsequently, 2.0 mL of a solution containing 20% sulfuric acid (*v*/*v*) and 0.1% titanium tetrachloride (*v*/*v*) was added to the 200 µL supernatant, followed by centrifugation. The supernatants were analyzed at 415 nm using an instrument to obtain the concentration of H_2_O_2_.

### 4.6. Determination of Antioxidant Enzyme Activities in Soybean Roots

Commonly analyzed antioxidant enzymes in roots include ascorbate peroxidase (APX), catalase (CAT), peroxidase (POD), and superoxide dismutase (SOD). The following kits were used for detection: APX Activity Assay Kit, CAT Activity Assay Kit, POD Activity Assay Kit, and SOD Activity Assay Kit (Shanghai Sangon Biotech, Shanghai, China). Briefly, 0.1 g of frozen sample was homogenized and extracted in PBS buffer. After centrifugation, the supernatant was collected via pipetting for enzyme activity determination. Subsequently, the activities of these enzymes were measured according to the manufacturer’s instructions. The activity of superoxide dismutase (SOD; EC 1.15.1.1) was measured using the total superoxide dismutase assay kit with WST-8. In the coupled reaction system of WST-8 and xanthine oxidase (XO), the SOD enzyme activity in the reaction system was defined as one unit of enzyme activity (U/mL) when the inhibition percentage reached 50%. The peroxidase (POD; EC 1.11.1.7) activity was measured by observing the oxidation of guaiacol after the addition of H_2_O_2_. One unit (U) of POD activity was defined as an increase of 0.5 in absorbance at 470 nm per minute per gram of root sample in the reaction system. Catalase (CAT; EC 1.11.1.6) activity was determined by monitoring the decrease in absorbance at 240 nm, indicating a decrease in H_2_O_2_ concentration. One unit (U) of CAT activity was defined as the degradation of 1 M H_2_O_2_ per minute per gram of root sample. The activity of the enzyme ascorbate peroxidase (APX; EC 1.11.1.11) was measured by monitoring the reduction in absorbance at 290 nm caused by the oxidation of ascorbic acid by the APX-H_2_O_2_ complex. The unit (U) of APX activity is defined as the oxidation of 1 M of ascorbate per minute per gram of root sample [47].

### 4.7. Determination of Citrate Synthase Activity and Citric Acid Content in Soybean Roots and Citric Acid Content in Treatment Solution

The following kits were used for detection: Citrate Synthase (CS) Activity Assay Kit (Shanghai Sangon Biotech, Shanghai, China), and Citric Acid (CA) Content Assay Kit (Shanghai Sangon Biotech, Shanghai, China). The activities of CS enzymes and the citric acid content, as well as the citric acid content in the treatment solution, were measured following the manufacturer’s instructions.

### 4.8. Statistical Analysis

Microsoft Office Excel 2019 and SigmaPlot 15.0 software were used to create all graphs and data analyses, and Photoshop CX6 was adopted for the drawing. One-way ANOVA and Duncan’s tests (*p* < 0.05) were performed to detect significant differences among the various treatments, denoted by distinct lower-case letters.

## 5. Conclusions

This study aimed to investigate the roles of B, Si, and their combination in alleviating Al toxicity in soybeans. The results revealed that the combined application significantly improved the growth and biomass of soybean roots exposed to Al toxicity compared to B and Si treatments alone. Our results indicated that Al toxicity causes PCD in soybean roots, while B, Si, and their combination all alleviated the PCD induced by Al toxicity. The administration of a combination of B and Si to soybeans resulted in a notable decrease in Al-induced oxidative stress, which was supported by the decreased levels of MAD and H_2_O_2_ production. Moreover, B, Si, and their combination significantly enhanced the plants’ antioxidant systems by up-regulating antioxidant enzyme activities (APX, CAT, POD, and SOD) compared to plants under Al stress alone. Overall, supplementation with B, Si, and their combination alleviated oxidative damage and reduced PCD induced by Al toxicity, which may be one of the mechanisms involved in alleviate root growth inhibition due to Al toxicity. However, the exact mechanisms underlying the regulatory roles of B and Si in the mechanisms of internal and external tolerance against Al toxicity in plants remain incompletely understood, and there are still many issues to be explored in future research: (1) How do Si, B and their complexes mitigate Al toxicity at the cellular and molecular levels in soybeans? (2) How do Si and B complexes interact to better mitigate the effects of Al toxicity on soybeans?

## Data Availability

Data is contained within the article.
